# Synthesis and Characterization of Silver Nanoparticles Stabilized with Biosurfactant and Application as an Antimicrobial Agent

**DOI:** 10.3390/microorganisms12091849

**Published:** 2024-09-06

**Authors:** Bruna G. A. Lima, Renata Raianny Silva, Hugo M. Meira, Italo J. B. Durval, Clovis Macedo Bezerra Filho, Thayse A. L. Silva, Leonie A. Sarubbo, Juliana Moura Luna

**Affiliations:** 1 Postgraduate Program in Development of Environmental Processes, Catholic University of Pernambuco (UNICAP), Príncipe Street, n. 526-Boa Vista, Recife 50050-900, Brazil; bruna.2021602028@unicap.br; 2 Northeast Biotechnology Network (RENORBIO), Federal Rural University of Pernambuco, Dom Manuel de Medeiros Street, Dois Irmãos, Recife 52171-900, Brazil; renatabiology2015@gmail.com; 3 Advanced Institute of Technology and Innovation (IATI), Potira Street, n. 31-Prado, Recife 50070-280, Brazil; hugo_morais15@hotmail.com (H.M.M.); italo.durval@iati.org.br (I.J.B.D.); leonie.sarubbo@unicap.br (L.A.S.); 4 School of Health and Life Sciences, Catholic University of Pernambuco (UNICAP), Príncipe Street, n. 526-Boa Vista, Recife 50050-900, Brazil; clovis.filho@unicap.br; 5 College of Chemical Engineering, State University of Campinas, São Paulo 13083-970, Brazil; thayse@unicamp.br; 6 Icam Tech School, Catholic University of Pernambuco (UNICAP), Príncipe Street, n. 526-Boa Vista, Recife 50050-900, Brazil

**Keywords:** silver nanoparticles, green synthesis, multiresistance, antimicrobial activity

## Abstract

Surfactants can be used as nanoparticle stabilizing agents. However, since synthetic surfactants are not economically viable and environmentally friendly, biosurfactants are emerging as a green alternative for the synthesis and stabilization of nanoparticles. Nanoparticles have been applied in several areas of industry, such as the production of biomedical and therapeutic components, packaging coating, solar energy generation and transmission and distribution of electrical energy, among others. The aim of this study was to synthesize, in a simple and green way, silver nanoparticles (AgNPs) using the biosurfactant produced by *Candida lipolytica* UCP 0899 as a stabilizer. AgNPs were examined and morphologically characterized using the techniques of ultraviolet–visible spectroscopy (UV–visible), scanning electron microscopy (SEM), zeta potential and energy dispersive X-ray spectroscopy (EDS). Newly formed silver nanoparticles showed a maximum UV–visible absorption peak at 400 nm, while a shift to 410 nm was observed in those stored for 120 days. SEM micrograph confirmed the formation of nanoparticles with an average size of 20 nm and with a predominant spherical structure, while a zeta potential of −60 mV suggested that the use of the biosurfactant promoted their stability. Stabilized nanoparticles were tested for their antimicrobial activity against bacterial isolates of *Pseudomonas aeruginosa*, *Staphylococcus aureus*, *Escherichia coli* and *Enterobacter* sp., as well as fungal isolates of *Candida albicans* and *Aspergillus niger*. At a concentration of 16.50 µg/mL, AgNPs inhibited the growth of all target microorganisms according to the following decreasing order: *E. coli* (95%), *S. aureus*, *C. albicans* (90%), *A. niger* (85%), *Enterobacter* sp. (75%) and *P. aeruginosa* (71%). These results suggest the potential use of the biosurfactant as a stabilizer of silver nanoparticles as an antimicrobial agent in different industrial sectors. Furthermore, the in vivo toxicity potential of biosurfactants was evaluated using the *Tenebrio molitor* model. The larvae were treated with concentrations in the range of 2.5, 5.0 and 10 g/L, and no mortality was observed within the 24 to 72 h period, demonstrating non-toxicity within the tested concentration range. These findings support the safety, efficacy and non-toxicity of biosurfactant-stabilized nanoparticles.

## 1. Introduction

Nanotechnology involves the manipulation and creation of new materials with a size below 100 nm. Nanomaterials (NMs) have biological, physical and chemical properties different from those of conventional-size materials, attracting the interest of several sectors, such as the pharmaceutical, electronics, textile, food, cosmetics and energy industries [1]. Research indicates that the global nanotechnology market could generate a profit of approximately 125 billion dollars by 2024 [2]. Among the nanomaterials under study, silver nanoparticles (AgNPs) play a prominent role due to their biological properties, such as antibacterial, antifungal, anti-inflammatory and antiviral activities [3]. In addition to biomedical applications, AgNPs are being investigated, for example, for use in products and processes related to the generation, transmission and distribution of electrical energy. These applications can bring technical and economic advantages to these installations [4].

Even though several methods of NM synthesis can be employed, the biological route or biosynthesis has aroused interest in preparing nanoparticles as a cheap, efficient and clean method using biocatalysts originating from bacteria, algae, filamentous fungi and plants as mediators [5].

The size and shape of nanoparticles are critical factors that greatly impact their biological activity and specificity. Since obtaining stable nanoparticles with controlled sizes through traditional chemical and physical processes still remains a challenge, surfactants are emerging as stabilizers [6]. However, synthetic surfactants are not economically viable and eco-friendly; therefore, biosurfactants may be a green alternative for nanoparticle synthesis and stabilization [7,8].

Biosurfactants produced by microorganisms, cultivated either in insoluble substrates (oils, residues and hydrocarbons) or in carbohydrate-containing soluble media, belong to different classes of biopolymers, such as glycolipids, lipopeptides, protein–polysaccharide complexes, phospholipids, fatty acids and neutral lipids, each one with its own properties and physiological functions [9].

Due to diverse structures and properties, biosurfactants find consolidated applications in various industrial processes, in addition to being proposed in many new applications [10]. Among these, they can be effectively employed in the production of nanomaterials, being able to easily form a variety of liquid crystals in aqueous solutions [8,11].

The synthesis of stable silver nanoparticles using surfactin [12] and a lipopeptide with fungicidal properties produced by *Bacillus cereus* [8] as stabilizers has been reported, while a rhamnolipid biosurfactant, consisting of a mixture of two rhamnolipids, was successful in the synthesis/stabilization of nanozirconia particles [13].

*Candida lipolytica,* also known as *Yarrowia lipolytica* [14], is among the most commonly studied yeasts for the production of biosurfactants for applications in various areas such as environmental remediation [15,16,17] and antimicrobial, antibiofilm and anti-adhesive activities against microorganisms [18,19]. Despite the rare cases of patients with potential susceptibility to *C. lipolytica* infection, this strain is considered a nonpathogenic microorganism and is classified as Generally Recognized as Safe (GRAS) by the Food and Drug Administration (USA). *C. lipolytica* species are capable of producing a variety of compounds of biotechnological interest, such as proteins, lipids and organic acids, and due to its metabolism, genetics and biotechnological importance, *C. lipolytica* has been extensively studied [14].

Therefore, the present study aimed to synthesize silver nanoparticles and stabilize them using the biosurfactant produced by *Candida lipolytica* UCP 0899. The nanoparticles obtained were examined and morphologically characterized through ultraviolet–visible spectroscopy (UV-Vis), scanning electron microscopy (SEM), zeta potential and energy dispersive X-ray spectroscopy (EDS), and, after stabilization with the biosurfactant, they were tested for their antimicrobial and antifungal potential against isolates of clinical origin.

## 2. Materials and Methods

### 2.1. Microorganism

*Candida lipolytica* UCP 0988, deposited in the Bank of Cultures of the Nucleus of Research in Environmental Sciences and Biotechnology (NPCIAMB) of the Catholic University of Pernambuco, was used as a biosurfactant producer.

### 2.2. Preparation of Inoculum

The yeast inoculum was standardized. The cultures were transferred to tubes with the YMA medium to obtain a young culture. Next, the samples were transferred to flasks with 50 mL of YMB medium, followed by incubation with constant stirring (150 rpm) at 28 °C for 24 h. Dilutions were then performed until reaching a concentration of 10^8^ cells/mL [20].

### 2.3. Biosurfactant Production

Fermentation for biosurfactant production was carried out in an aqueous medium (pH 5.5) containing 4% molasses, 2.5% corn steep liquor and 2.5% residual soybean oil. The inoculum (1% *v*/*v*) was added to the medium up to a concentration of 10^8^ cells/mL. The flasks were incubated in a shaker under agitation at 200 rpm for 144 h at 28 °C [20].

### 2.4. Biosurfactant Isolation

The biosurfactant was extracted from the fermented broth after removal of cells by centrifugation (5000× *g*, 15 min, 4 °C). For this purpose, the same volume of ethyl acetate (1:1, *v*/*v*) was added to the cell-free broth. The mixture was vigorously stirred for 15 min and allowed to stand to separate the phases. The samples were extracted twice, and the organic phase was evaporated at 40 °C to remove the solvent. The residue obtained was washed twice with hexane to remove any remaining hydrophobic substances, such as fatty acids and alcohols resulting from fermentation. After extraction, the product was treated with a base and crystallized for maximum removal of impurities [21].

### 2.5. Synthesis of Silver Nanoparticles

All reagents were of analytical grade and used without additional purification. The technique used to prepare silver nanoparticles (AgNPs) was adapted from Le et al. [22]. Firstly, 1.7 g (1.0 × 10^−2^ mol) of silver nitrate (AgNO_3_) was dissolved in 100 mL of deionized water, and the resulting solution was precipitated with 0.62 g (1.55 × 10^−2^ mol) of sodium hydroxide. The precipitate, composed of Ag_2_O, was filtered and dissolved in 100 mL of aqueous ammonia (NH_3_) (0.4% *w*/*w*, 2.3 × 10^−2^ mol) until the formation of a clear solution of silver–ammonia complex, [Ag(NH_3_)_2_]^+^. Then, 2.5 g of the isolated biosurfactant (BS) was added to the complex, and the resulting solution was gently stirred for 2 h at 25 ± 0.5 °C until obtaining a homogeneous mixture. Finally, 2 g (1.11 × 10^−2^ mol) of glucose was added to the mixture at room temperature under gentle stirring. The reduction of the silver complex solution was carried out in quartz glass under ultraviolet (UV) light irradiation and vigorous stirring without heating. A UV lamp (λ = 365 nm, 35 W) was used as a light source. After 8 h of irradiation, a transparent dispersion of AgNPs stabilized with biosurfactant was obtained. The synthesis of AgNPs was successfully conducted with a final silver concentration of around 1%. The obtained dispersion containing silver nanoparticles complexed with biosurfactant (BS-AgNPs) was stored at 4 °C for further experiments.

### 2.6. Characterization of Silver Nanoparticles

The absorption properties of the BS-AgNP dispersion were analyzed using a UV-Vis spectrophotometer (model Espec-UV-5100, Tecnal, Piracicaba, SP, Brazil) in the wavelength range between 320 and 450 nm. The zeta potential and, consequently, the stability of BS-AgNPs in the dispersion were determined using a particle analyzer (model 4.0+ ZM3-D-G Direct Imaging equipment, Zeta Meter, Harrisonburg, VA, USA) based on Laser Doppler Electrophoresis and operating on Dynamic Light Scattering.

### 2.7. Scanning Electron Microscopy and Energy Dispersive X-ray Spectroscopy Analyses

SEM was used to examine the morphology of nanoparticles, and EDS was used to make a semiquantitative evaluation of the chemical elements existing on the surface of the sample. SEM analyses were performed with a scanning electron microscope (model Mira 3, Tescan USA, Warrendale, PA, USA) using an accelerating voltage of 30 kV. The scanning electron microscope coupled with an EDS detector (model 40 Ultim Max, Oxford Instruments, Abingdon, UK) was used to carry out the EDS analyses. The nanoparticles were deposited on the surface of a carbon ribbon at a concentration of 0.01 mg/mL (diluted in ultrapure water), subsequently covered with a Petri dish and dried at room temperature; finally, a thin layer of gold coating was created to make the samples conductive.

### 2.8. Determination of Antimicrobial Activity of Silver Nanoparticles Stabilized with Biosurfactant

To determine the antimicrobial activity of BS-AgNPs, *Pseudomonas aeruginosa*, *Enterobacter* sp., *Escherichia coli*, *Staphylococcus aureus*, *Aspergillus niger* and *Candida albicans* strains were collected from clinical isolates from a reference state hospital, as the target microorganisms. In the study, the Kirby–Bauer disk diffusion method was used to assess the antibacterial effect [23]. Briefly, 100 µL of a bacterial suspension (5 × 10^6^ CFU/mL) was evenly spread over the surface of a nutrient agar plate. Then, filter paper discs (about 6 mm in diameter) impregnated with BS-AgNPs (16.50 μg/L) were placed on the agar surface. The agar plates were then inverted and incubated at 37 °C for 24 h, and the diameter of the zone of inhibition around the disk was measured with a ruler. The antifungal activity was determined using Potato Dextrose Agar (PDA) fungal growth medium with the addition of 200 μL of BS-AgNPs dispersion up to a concentration of 16.50 μg/L per Petri dish, according to the method described by Durval et al. [8]. After preparing the plates, the fungi were inoculated, the plates were incubated at 30 °C for 48 h, and the size of the inhibition halos was recorded.

### 2.9. Biosurfactant Toxicity to Tenebrio molitor

*Tenebrio molitor* larvae, each approximately 100 mg, were randomly assigned to groups of at least 10 individuals. A volume of 10 µL of biosurfactant was administered using a Hamilton syringe at concentrations of 2.5, 5.0 and 10 g/L. Larval viability was assessed at 24, 48, 72 and 96 h, determined by the absence of movement. Larvae inoculated with PBS served as negative controls. The results were plotted as survival curves over time, according to Silva et al. [24].

## 3. Results and Discussion

### 3.1. Characterization of Silver Nanoparticles

**Figure 1:**
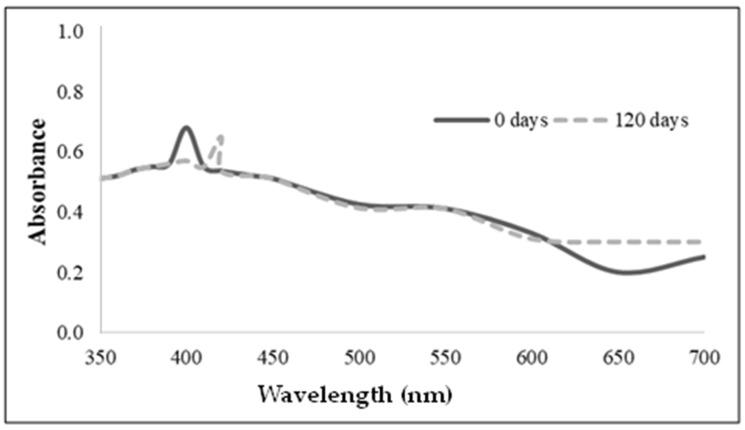
A UV-visible spectrum of the nanoparticles synthesized with the *Candida lipolytica* biosurfactant (BS-AgNPs) just after their preparation (0 days) and after 120 days.

The maximum absorption peak at 400 nm appearing in the UV-Vis spectrum of the newly synthesized silver nanoparticles stabilized with the *C. lipolytica* biosurfactant (Figure 1) indicates the actual formation of nanoparticles. This spectral characteristic, in fact, almost corresponds to that observed for AgNPs containing plant material (410 nm) or wrapped by rhamnolipid biosurfactant (400 nm) using Localized Surface Plasmon Resonance (LSPR), which can be considered typical of silver nanoparticles. In this sense, Tyagi et al. [25] suggested that nanoscale silver can be synthesized in reverse micelles used as a stabilizer.

**Figure 2:**
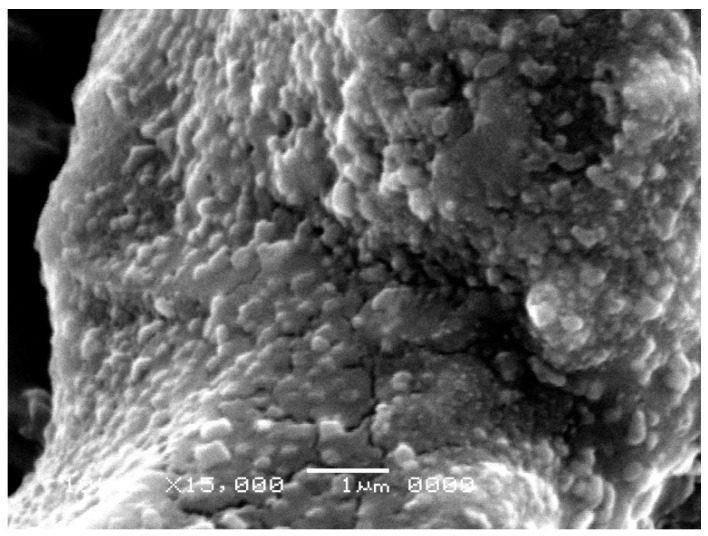
Scanning electron microscopy (SEM) image: silver nanoparticles stabilized by the *Candida lipolytica* biosurfactant.

Figure 2 shows the SEM micrographs of the biosurfactant and silver nanoparticles stabilized by the *C. lipolytica* biosurfactant. The synthesized BS-AgNPs appeared to be stable and spherical, likely because the biosurfactant acted as a stabilizing agent, preventing the formation of aggregates. Additionally, nanoparticles predominantly exhibited an average diameter of approximately 20 nm, as confirmed by post-synthesis spectrophotometry (Figure 1). However, larger sizes were observed under microscopy, possibly due to manipulation and storage after synthesis.

A comparison can be made with the results of similar nanoparticles reported in the literature. Immediately after synthesis, the BS-AgNPs prepared by Elakkiya et al. [26] using a *P. aeruginosa* TEN01 rhamnolipid biosurfactant showed a spherical shape and a diameter (around 20 nm) practically coincident with that observed in the present study. Xie et al. [27] reported a size in the 2–8 nm range for BS-AgNPs stabilized with a similar commercial rhamnolipid biosurfactant (Jeneil Biosurfactant Co. LLC, Saukville, WI, USA) in heptane. Durval et al. [8], who prepared BS-AgNPs stabilized with a *B. cereus* UCP 1615 biosurfactant, observed similar high-density structures but with larger diameters (30–150 nm), in addition to a random particle distribution. In addition, Kiran et al. [28] used a glycolipid biosurfactant produced by the marine *Brevibacterium casei* MSA19 strain using agro-industrial and industrial waste as a substrate to synthesize BS-AgNPs, which proved to be uniform and stable for 2 months. After 120 days of storage, BS-AgNPs exhibited a plasmonic absorption peak at a wavelength (420 nm) close to that observed in this study, which indicated an increased particle size and stabilization by the biosurfactant. As for the use of synthetic surfactants as stabilizers, Soukupová et al. reported the synthesis of AgNPs with sizes in the 59–70, 43–53 and 57–76 nm ranges using cationic cetyltrimethylammonium chloride (CTAC), anionic sodium dodecyl sulfate (SDS) and non-ionic Tween 80 surfactants, respectively [29].

**Figure 3:**
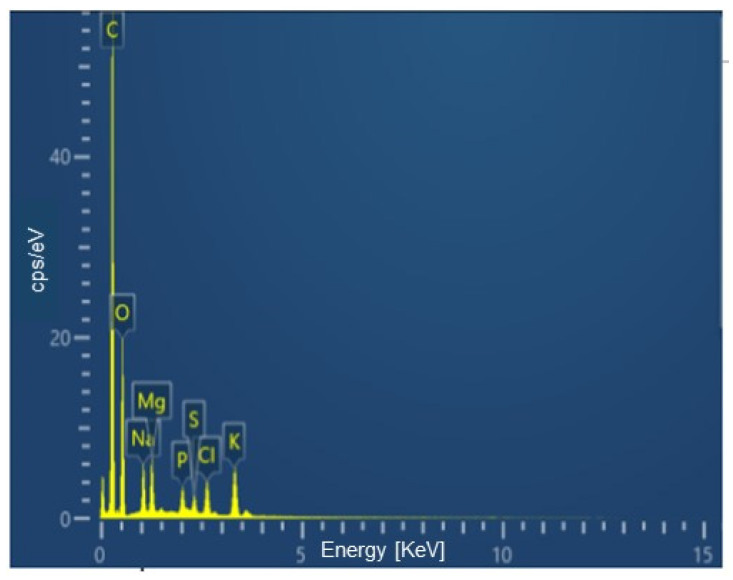
EDS analysis of the biosurfactant produced by *Candida lipolytica*.

**Figure 4:**
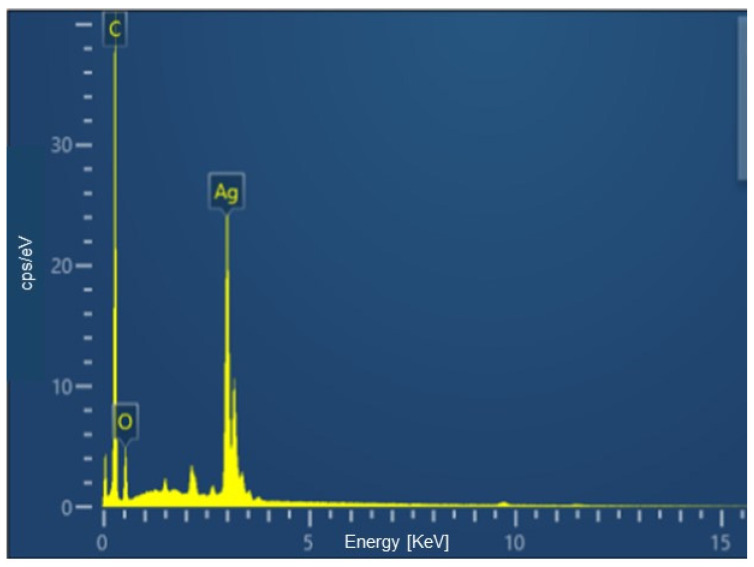
EDS analysis of the silver nanoparticles stabilized by the *Candida lipolytica* biosurfactant.

Through EDS, an analytical technique that allows the elemental analysis of materials, it was possible to quantify the percentages of the main elements constituting both the biosurfactant and BS-AgNP. These results are illustrated in Figure 3 and Figure 4, respectively. The data obtained are another indication of the actual formation of nanoparticles since the element silver appeared in the BS-AgNP spectrum as a result of its attractive interaction with the biosurfactant. The carbon shown is from the carbon chain of the biosurfactant used to synthesize the silver nanoparticles.

The zeta potential, which reflects the surface potential of particles, is influenced by changes in the interface with the dispersion medium due to the dissociation of functional groups on the particle surface or the adsorption of ionic species. This parameter, which is usually determined with particle analyzers based on electrophoresis techniques, expresses the effective charge of particles and depends on the electrostatic repulsion between them and the dispersion stability [30].

The zeta potential of BS-AgNPs (−60.6 ± 1.2 mV) was sufficiently high (in absolute value) to indicate strong electrostatic repulsion among particles, resulting in a low tendency to aggregation or precipitation and, therefore, in high colloidal stability.

Lower colloidal stability can be deduced for similar BS-AgNPs described in the literature. Zeta potential values in the range of −23.4 ± 1.4 mV, −38.7 mV and −31.6 mV were, in fact, measured for BS-AgNPs prepared using the following stabilizers: (a) a biosurfactant produced by *B. cereus* UCP 1615 [8], (b) a lipopeptide-rich cell-free extract from *Bacillus paramycoides* fermentation in an olive oil-supplemented medium [31], and (c) a rhamnolipid produced by *P. aeruginosa* [26], respectively. Another study [32] carried out with the same *P. aeruginosa* rhamnolipid demonstrated the role of the biosurfactant. In this study, the colloidal stability of BS-AgNPs prepared using AgNO_3_ at pH 12 was remarkably enhanced, which led to a zeta potential increase (in absolute value) from −23.8 mV to −56.3 mV [32].

### 3.2. Determination of Antimicrobial Activity

Bacterial resistance, according to the World Health Organization (WHO), is one of the main public health threats in the world in the 21st century due to the great difficulty in effectively treating pathologies caused by bacteria and fungi of clinical origin, consequently leading to an increase in public health expenditure due to the extension of treatment doses and hospitalizations. Another problem generated primarily by microorganisms is biofouling, which affects different systems such as water purification plants, turbines and heat exchangers, marine equipment, biomedical devices, food packaging, textiles and historical materials [33]. For these reasons, the present work evaluated the bactericidal and fungicidal potential of BS-AgNPs against *E. coli*, *P. aeruginosa*, *Enterobacter* sp., *A. niger*, *S. aureus* and *C. albicans*.

Table 1 shows the antimicrobial activity, from which it can be seen that BS-AgNPs have great potential to inhibit the growth of the selected target microorganisms.

At a concentration of 16.50 µg/mL, BS-AgNPs were able to almost completely suppress the growth of the Gram-positive bacterium *S. aureus* (by 90%) and the Gram-negative bacterium *E. coli* (by 95%) and also satisfactorily inhibit the Gram-negative bacteria *Enterobacter* sp. (by 75%) and *P. aeruginosa* (by 71%). Gram-negative bacteria are generally more resistant to antimicrobial agents than Gram-positive ones [34] because their lipopolysaccharide-containing outer cell membrane ends up hindering the entry and, consequently, the diffusion of AgNPs into these microorganisms. Nonetheless, the surprising result obtained against *E. coli* is consistent with that reported by Anthony et al. [35], who observed complete suppression of *E. coli* growth in liquid Mueller-Hinton Broth at 10 μg/mL AgNPs dosage.

Yu et al. [36] observed that AgNPs synthesized with the antibacterial lipopeptide from *B. subtilis* SDUM301120 were able to inhibit the growth of *E. coli* ATCC 25,922 and *S. aureus* ATCC 29,213 strains at concentrations of 8.1 and 8.3 μg/mL, respectively, while Bezza at al. [37], using a different technique and another strain of *B. subtilis* (CN2), observed inhibition of *P. aeruginosa* growth with 15.6 μg/mL of AgNP. According to Sivasubramanian et al. [38], when produced using the *Cassia alata* leaf extract, it inhibited the growth of *Enterobacter* sp. with 6.86 μg/mL.

Regarding fungi, it was observed that BS-AgNPs were able to almost completely suppress the growth of the yeast *C. albicans* (90%) and, to an intermediate extent, also that of the mold *A. niger* (85%). Durval et al. [8] observed, using BS-AgNPs stabilized with a biosurfactant produced by *B. cereus*, 85.78% inhibition of *Aspergillus niger* growth at the highest concentration (16.50 μg/mL) and 74.20% inhibition at the lowest one (1.65 μg/mL). These results highlight the importance of BS-AgNP concentration on antimicrobial activity. AgNPs biosynthesized from a cell filtrate of *Pseudomonas fluorescens* were able to inhibit *A. niger* growth only at a concentration of 150 μg/mL [39]. Silver nanoparticles obtained from *Aspergillus flavo-furcatis* showed antimicrobial activity by the agar well diffusion method against *C. albicans*, *E. coli* and *S. aureus*, with zones of inhibition ranging from 12.0 to 19.8 mm [40]. The study of Hassan et al. [41] demonstrated that AgNPs synthesized from a cell-free filtrate of *Aspergillus flavus* culture inhibited the growth of *C. albicans*, *E. coli* and *S. aureus* with inhibition halos of 18.33, 13.33 and 15.00 mm, respectively. Finally, Rani et al. [42] observed that the AgNPs prepared using the filtrate of *Aspergillus terreus* Thom 1918 inhibited the growth of both *E. coli* and *S. aureus* at concentrations of 11.43 and 32.2 μg/mL, respectively.

Data from the literature have confirmed the strong activity of AgNPs against Gram-positive and Gram-negative bacteria. In addition, they are also considered as potential biocompatible and economic antifungal agents. The antimicrobial effect of AgNPs and related nanomaterials is evidenced, which is essential due to the growing microbial resistance to antibiotics, in addition to the effectiveness in prophylaxis of bacterial colonization of prostheses and catheters [43,44,45,46].

The results described in the literature corroborate the data obtained in this work, thus indicating the potential of nanoparticles as antimicrobial agents, with the benefit of having been obtained through a green method using a biocompatible stabilizing agent, the biosurfactant.

### 3.3. Biosurfactant Toxicity to Tenebrio molitor

**Figure 5:**
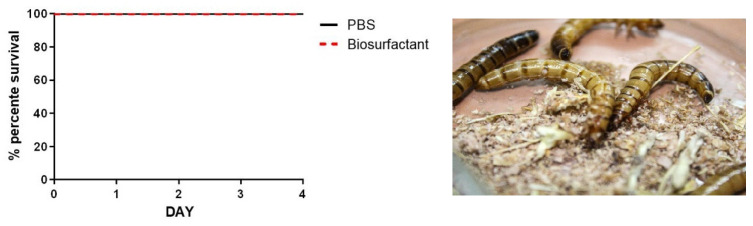
Effects of biosurfactant toxicity to *Tenebrio molitor* larvae treated with phosphate-buffered saline (PBS), used as a negative control.

The results showed no significant difference in the survival rates between the groups treated with biosurfactants and the control group (PBS-treated). This suggests that biosurfactants at the tested concentrations are safe for *Tenebrio molitor* larvae, as they did not present any evident toxic effect in a simple and cost-effective model [47]. The survival curves over time support this observation (Figure 5), showing a high viability rate of larvae in all groups. These findings are significant as they suggest the potential use of biosurfactants in applications where safety and non-toxicity are essential.

## 4. Conclusions

The biosurfactant produced by *Candida lipolytica* UCP 0899 in a low-cost medium supplemented with industrial waste proved to be efficient as a stabilizing agent in the green synthesis of silver nanoparticles (BS-AgNPs). The BS-AgNPs obtained showed stability over time and were small, spherical and uniform, being compatible with several biotechnological applications. The nanoparticles exhibited antimicrobial activity against pathogens that harm human health. The *Tenebrio molitor* model proved to be fast, cheap, painless and easy to execute, demonstrating the high viability and safety of the tested biosurfactant. Therefore, it can be concluded that biosurfactants have the potential to be used as eco-friendly stabilizers of nanoparticles in productions based on green synthesis. The global market for nanotechnology is becoming increasingly competitive due to its potential applications in various industrial segments that are not necessarily related to the biological properties of nanoparticles.

## Figures and Tables

**Table 1:** Rate of growth inhibition (%) caused by BS-AgNPs at a concentration of 16.50 µg/mL against different bacteria and fungi. Data expressed as mean plus standard deviation of three independent analyses.

Microorganism	Rate of Growth Inhibition (%)
*Pseudomonas aeruginosa*	71 ± 0.3
*Enterobacter* sp.	75 ± 0.4
*Aspergillus niger*	85 ± 0.3
*Escherichia coli*	95 ± 0.5
*Staphylococcus aureus*	90 ± 0.2
*Candida albicans*	90 ± 0.1

## Data Availability

Data are contained within the article.
